# Quality-of-life Evaluation of Healthy Siblings of Children with Chronic Illness

**DOI:** 10.4274/balkanmedj.galenos.2019.2019.7.142

**Published:** 2019-12-20

**Authors:** Meltem Dinleyici, Kürşat Bora Çarman, Canan Özdemir, Koray Harmancı, Makbule Eren, Birgül Kirel, Enver Şimşek, Coşkun Yarar, Aysu Duyan Çamurdan, Figen Şahin Dağlı

**Affiliations:** 1 Department of Social Pediatrics, Eskişehir Osmangazi University School of Medicine, Eskişehir, Turkey; 2 Department of Pediatric Neurology, Eskişehir Osmangazi University School of Medicine, Eskişehir, Turkey; 3 Department of Pediatric Hematology and Oncology, Eskişehir Osmangazi University School of Medicine, Eskişehir, Turkey; 4 Department of Pediatric Allergy and Immunology, Eskişehir Osmangazi University School of Medicine, Eskişehir, Turkey; 5 Department of Pediatric Gastroenterology and Hepatology, Eskişehir Osmangazi University School of Medicine, Eskişehir, Turkey; 6 Department of Pediatric Endocrinology, Eskişehir Osmangazi University School of Medicine, Eskişehir, Turkey; 7 Department of Social Pediatrics, Gazi University School of Medicine, Ankara, Turkey

**Keywords:** Children, chronic disease, quality of life, sibling

## Abstract

**Background:**

Chronic disease of children can cause changes in the health-related quality of life (HrQoL) of the family members.

**Aims:**

To evaluate the HrQoL of healthy siblings of children with chronic disease.

**Study Design:**

Cross-sectional study.

**Methods:**

The study included healthy sibling of children with chronic disease (cerebral palsy, epilepsy, diabetes, celiac disease, hematologic/oncologic disease, or asthma) and healthy sibling of healthy children to evaluate the quality of life. We used the Pediatric Quality of Life Inventory questionnaire; the physical health and psychosocial health scores were calculated using the responses of the sibling and parent. The primary endpoint was the comparison of HrQoL scores of healthy siblings of children with chronic disease and that of healthy siblings of healthy children.

**Results:**

This study included a respective healthy sibling of 191 children with chronic disease and healthy sibling of 100 healthy children. The physical health, psychosocial health, and total health scores of healthy siblings of children with chronic disease were significantly lower than that of healthy siblings of healthy children (p<0.001). Among the healthy siblings of children with chronic disease, the lowest psychosocial health score was found in the siblings of children with cerebral palsy, hematologic/oncologic disease, and asthma (p<0.001). The global impact on the quality of life for healthy siblings of children with chronic disease was significantly higher in the self-report of the children than that of the parents (30.4% versus 15.1%, p<0.05).

**Conclusion:**

Most healthy siblings of children with chronic disease are physically and psychosocially affected and there is low parental awareness of this condition. This can increase the risk of emotional neglect and abuse of these children. Therefore, special support programs are needed for the families of children with chronic diseases.

Chronic disease is defined as a long-term, untreatable condition or current symptoms that limit activities of daily life (1). The diagnosis of a chronic physical condition in a child may cause serious stress in the family, which may lead to changes in the family structure and result in labor loss (2). It also has psychosocial impact on the siblings of the children with chronic disease (3). The development of depression and anxiety was more common in the siblings of children with chronic disease than the healthy children; these children also showed less communication with their peers. Consequently, they have an increased risk of internalizing problems and developing a negative self-concept (3,4).

The pediatric quality of life inventory (PedsQL) is a tool for measuring the health-related quality of life (HrQoL) in children. The PedsQL 4.0 Measurement Model is a multidimensional questionnaire consisting of physical, emotional, social, and school functioning domains (5). Quality of life (QoL) questionnaires can be given to an ill child, the sibling and parents of the ill child, and/or healthy children (4). As of 2019, six studies have used the PedsQL to evaluate the HrQoL of siblings of children with chronic disease (6,7,8,9,10,11). In the studies conducted so far, the number of participants ranged from 10 to 131, with a mean age of 10 to 13 years; the chronic diseasees that have been studied included mainly epilepsy, cancer, type 1 diabetes, and cystic fibrosis (4,11). The studies were conducted in Canada, Belgium, the United States, India, and the United Kingdom (4,11). However, no study has been conducted on the siblings of children with chronic disease in Turkey. The HrQoL assessment of siblings of children with chronic disease allows the assessment of the increased functional loss caused by the specific type of disease and provide guidance on the strategies to reduce the negative impact on the siblings at high risk (12).

In this study, we aimed to evaluate the HrQoL scales of siblings of children with chronic disease and compare with those of siblings of healthy children. We also aimed to compare the HrQoL scores of children with different chronic diseasees. Based on the HrQoL assessment, we aimed to gain insight into the preventive and supportive strategies for patients with an impaired QoL.

## MATERIALS AND METHODS

This case-control study was carried out in the inpatient and outpatient services of the Eskişehir Osmangazi University School of Medicine between May 2016 and May 2017. The study was approved by the Eskişehir Osmangazi University Institutional Ethics Committee for Non-Interventional Clinical Trials (decision no. 14 dated 18 April 2016).

### Inclusion criteria

1. Children, aged 2 to 18 years, who were diagnosed with chronic disease (such as hematologic/oncologic malignancy, type 1 diabetes, celiac disease, epilepsy, cerebral palsy, and asthma) ≥3 months before the study, or healthy children aged 2 to 18 years without any chronic condition and need for regular use of medications.

2. Healthy siblings of children diagnosed with chronic disease ≥3 months before the study (chronic disease group) and healthy siblings of healthy children without any chronic condition and need for regular use of medications.

### Primary and secondary endpoints

The primary endpoint of the study was to evaluate the HrQoL scores of the healthy siblings of children with chronic physical disease and to compare with those of the siblings of healthy children. Secondary endpoints were as follows: (1) to compare the HrQoL scores of the siblings of children with chronic physical disease based on the type of disease and to compare their scores with those of the healthy siblings of healthy children, (2) to evaluate the HrQoL scores of the siblings of children with chronic physical disease based on the type of disease and gender, and (3) to evaluate the HrQoL scores of the siblings of children with chronic physical disease based on the type of disease and age of healthy sibling.

We collected data after obtaining consent from the parents and siblings of the children with chronic disease. The demographic characteristics of the children with chronic condition, as well as of their parents and siblings, were recorded on the questionnaire. The PedsQL was given to the parents and siblings who were of an appropriate age to complete a questionnaire. The validity and reliability of the Turkish translation of the PedsQL inventory have already been established. Written permission to use the tool was granted by James W. Varni, who developed the tool.

Based on the age group of the siblings of children with chronic disease, one of the following forms was used: PedsQL Child Form (2-4 years), PedsQL Child Form (5-7 years), PedsQL Child Form (8-12 years), and PedsQL Adolescent Form (13-18 years). The PedsQL 4.0 consists of 23 questions that cover four domains of HrQoL measurement in children. Two separate forms, namely the child and parent forms, were completed for all children who participated in the study. We used a 3-response and 5-response visual Likert scale for children aged <8 years and those aged 8-18 years, respectively. The physical health score was based on the mean score of eight questions on physical functioning; the responses were presented as 100 (never), 75 (seldom), 50 (sometimes), 25 (often), and 0 (almost always). The psychosocial health score was based on the mean score of three different categories: emotional functioning, social functioning, and school functioning. The total health score was based on the summary of score across all four categories of functioning and ranged from 0 to 100, with the higher scores indicating a better QoL. The Child and Adolescent Form of the PedsQL was used to evaluate the past one month of the child or adolescent (13,14,15).

### Statistical analysis

Based on sample size calculation, each group needs to include ≥ 84 children (a power of 0.8, on the basis of 4 predictors; the probability level was 0.05). For potential subgroup analysis of children with chronic disease, we enrolled 2:1 (chronic disease group: control group) and we increased 20% for potential dropouts; overall, there were 200 children in the chronic disease group and 100 in the control group). Statistical analysis was performed using the SPSS software (Chicago, IL) for Windows version 11.5. The normality of the demographic data and PedsQL scores was analyzed using the Shapiro-Wilks test; this test showed that the PedsQL scores were normally distributed. The results were summarized as mean and standard deviation. Demographic and categorical data were presented as percentages; the chi-square and Student t test were used for analyzing the statistical significance between two group. Analysis of variance (ANOVA) was used to analyze the QoL scores in the chronic disease group. Since the QoL scores were not homogeneously distributed, the Games-Howell test was used to analyze the subgroups when there was a statistically significant difference between the groups. A p value <0.05 was considered statistically significant.

## RESULTS

We enrolled the a respective sibling of 200 children with chronic disease. Of these, nine children were excluded because they did not respond to >50% of the questions. Therefore, a total of 191 healthy siblings of 191 children with chronic disease were finally evaluated. The control group included age-matched 100 healthy children with a respective healthy sibling.

### Demographic characteristics

There was no statistically significant difference in the age, educational level, and socioeconomic level of the parents in the chronic disease group and control group (p>0.05). The monthly income of the parents of the participants were evaluated and were categorized into low-, middle-, and high-income groups based on the country-specific data. Within the chronic disease group, 11% of the parents were in the low-income group, 82% were in the middle-income group, and 7% were in the high-income group; within the control group, 8% of the parents were in the low-income group, 84% were in the middle-income group, and 8% were in the high-income group. No statistically significant difference was found in the educational level and monthly income of parents of the chronic disease group and the control group (p>0.05).

Of the 191 children with chronic disease, 85 were boys and 106 were girls; the mean age was 116±56 months. There were 26 children with cerebral palsy, 39 with hematologic/oncologic disease, 31 with asthma, 28 with diabetes, 33 with celiac disease, and 34 with epilepsy. The median duration from the diagnosis of chronic disease to the enrollment in the study was 36 months. The median duration from the diagnosis cerebral palsy, hematologic/oncologic disease, and asthma, the epilepsy, the diabetes, and celiac disease to the enrollment in the study was 72, 24, 36, and 48 months, respectively. The duration from the diagnosis cerebral palsy to the enrollment in the study was greater than that of epilepsy (p<0.05) and asthma (p<0.05); there was not significant difference with respect to hematologic/oncologic disease, diabetes, and celiac disease (p>0.05).

There was no significant difference in the age and gender of the healthy siblings of children in the chronic disease group and the healthy siblings of the children in the control group (p>0.05; Table 1. Of the 191 healthy siblings of children with chronic disease, there were 102 boys and 89 girls; the mean age was 119±58 months. Overall, 101 (52.9%) healthy siblings of children with chronic disease were older than the index case. The median percentage of healthy siblings of the children with cerebral palsy, hematologic/oncologic disease, asthma, diabetes, celiac disease, and epilepsy who were older than the index case was 61.5%, 35.9%, 45.2%, 42.9%, 45.5%, and 55.9% respectively; there was no statistically significant difference between these disease subgroups (p>0.05) (Table 1).

### Self-reports of healthy siblings

The psychosocial health, physical health, and total health scores of the healthy siblings of children in the chronic disease group were significantly lower than that of the healthy siblings of the children in the control group (p<0.001, p<0.01, and p<0.001, respectively; Table 2). The mean psychosocial health score of the HrQoL scale was 72.6±13.7 among the healthy siblings of children in the chronic disease group versus 82.1±11.6 among the healthy siblings of children in the control group (p<0.001). The mean physical health score was lower in the the healthy children in the chronic disease group than that in the control group (82.6±13.8 versus 87.2±8.8, respectively, p<0.01). The total PedsQL score (psychosocial and physical health scores combined) of the healthy siblings of children in the chronic disease group was also significantly lower than that of the healthy siblings of the children in the control group (75.1±11.6 versus 83.4±10.4, respectively, p<0.001; Table 2).

Based on the self-report of the healthy siblings, the psychosocial health scores were significantly lower among the healthy siblings of the children with cerebral palsy, hematologic/oncologic disease, and asthma than that of the healthy siblings of the children in the control group (p<0.05) (Table 3); there was no significant difference between the healthy siblings of the children in the chronic disease group and that of healthy siblings of children in the control group in terms of diabetes, celiac disease, and epilepsy (p>0.05). The psychosocial health scores of healthy siblings of children with cerebral palsy, hematologic/oncologic disease, and asthma were lower than that of healthy siblings of children with celiac disease (p<0.05, p<0.001, and p<0.01, respectively). Similarly, the psychosocial health score of healthy siblings of children with cerebral palsy was lower than that of healthy siblings of children with diabetes (p<0.01) (Table 3).

Based on the self-report of the healthy siblings, the physical health scores of healthy siblings of the children with hematologic/oncologic disease were significantly lower than that of healthy siblings of the children in the control group (p<0.05); there was no significant difference between the healthy siblings of the children in the chronic disease group and those of children in the control group in terms of asthma, diabetes, celiac disease, epilepsy, and cerebral palsy (p>0.05).

Based on the self-report of the healthy sibling, the total QoL scores of the healthy siblings of the children with hematologic/oncologic disease, asthma, and cerebral palsy were significantly lower than that of healthy siblings of the children in the control group (p<0.001, p<0.05, and p<0.05, respectively). The total QoL score of children with a hematologic/oncologic disease was lower than that of children with diabetes and celiac disease (p<0.01 and p<0.001, respectively); in contrast, the total QoL score of children with asthma was lower than that of children with celiac disease (p<0.05; Table 3). When the psychosocial health, physical health, and total scores of the siblings of children in the chronic disease group were evaluated (from the perspective of the healthy sibling or parents), there was no correlation with the duration of chronic disease, irrespective of the type of disease (p>0.05) (Table 3).

### Parents’ report on the healthy siblings

The summary of QoL scores of the healthy siblings of children in the chronic disease group and control group was higher in the parents’ report than that in the healthy siblings’ self-report (p<0.05). The psychosocial health, physical health, and total scores of the siblings of children in the chronic disease group were significantly lower than that of the healthy siblings of children in the control group (p<0.001; Table 4). The mean psychosocial health score of the healthy siblings of children in the chronic disease group was 76.8±12.3 and that of the healthy siblings of children in the control group was 88.1±16.6 (p<0.001). The mean physical health score was lower in the chronic disease group than that in the control group (80.7±15.4 versus 87.6±8.5, p<0.001). The total PedsQL score of the healthy siblings of children with chronic disease was significantly lower than that of the children in the control group (77.7±11.3 versus 88.0±6.0, p<0.001; Table 4).

Based on the parents report on healthy siblings, the psychosocial health score of the healthy siblings of the children with cerebral palsy (p<0.01), hematologic/oncologic disease (p<0.001), asthma (p<0.001), diabetes (p<0.001), celiac disease (p<0.001), and epilepsy (p<0.001) was lower than that of the healthy siblings of children in the control group (Table 5). We found no statistically significant difference in the psychosocial health score between the healthy siblings of the children in the chronic disease group and that of the healthy siblings of children in the control group when the different chronic diseases were compared with each other (p>0.05) (Table 5).

Based on the parents’ report on healthy siblings, the physical health score of the healthy siblings of the children in with hematologic/oncologic diseases and diabetes was significantly lower than that of the healthy siblings of children in the control group (p<0.05); in contrast, there was no significant difference between the healthy siblings of the children with cerebral palsy, asthma, celiac disease, and epilepsy and the healthy siblings of the children in the control group (p>0.05). We found no statistically significant difference in the physical health score of the healthy siblings of the children in the chronic disease group and that healthy siblings of children in the control group when the different chronic diseases were compared with each other (p>0.05).

Based on the parents’ report on healthy siblings, the total QoL score of healthy siblings of the children with cerebral palsy (p<0.01), hematologic/oncologic diseases (p<0.001), asthma (p<0.001), diabetes (p<0.001), celiac disease (p<0.01), and epilepsy (p<0.01) was significantly lower than that of the healthy siblings of the children in the control group. We found no statistically significant difference in the total QoL score of the healthy siblings of the children in the chronic disease group and that of the healthy siblings of children in the control group when the different chronic diseases were compared with each other (p>0.05; Table 5). There was no correlation between the psychosocial health, physical health, and total health scores of the healthy siblings of children with chronic disease (in the responses of healthy siblings and parents) and the duration of the chronic disease (p>0.05).

### Comparison of psychosocial health, physical health, and total health scores based on gender

Based on the self-report of the healthy sibling of children with chronic disease, we found no significant difference in the psychosocial health, physical health, and total health scores between boys and girls (p>0.05). Similarly, based on the parents’ report on children with chronic disease, we found no significant difference in the psychosocial health, physical health, and total health scores between boys and girls (p>0.05).

### Comparison of psychosocial health, physical health, and total health scores by age of healthy siblings

Among the healthy siblings of children with chronic disease, 101 (52.9%) were older than the index case. Based on their self-report, there was no significant difference in the psychosocial health, physical health, and total health scores between the healthy siblings who were older and those who were younger than the respective ill children (p>0.05). Similarly, based on the parents’ report of children with chronic disease, there was no significant difference in the psychosocial health, physical health, and total health scores of the healthy siblings who were older and those who were younger than the respective ill children (p>0.05).

### Prevalence of impaired QoL in healthy siblings of children with chronic disease

In the present study, the self-report of the healthy siblings showed that impaired QoL was higher among the healthy siblings of children in the chronic disease group than that of healthy siblings of children in the control group (30.4% versus 14.1%, respectively, p=0.005). The parents’ report showed no impairment in the QoL of the healthy siblings of the children in the control group, whereas 15.1% of the healthy siblings of the children in the chronic disease group had an impaired QoL (p<0.001). Thus, the parents reported less impairment than the healthy siblings (30.4% versus 15.1%, respectively, p<0.05), indicating that parents had a poor awareness of the impaired QoL of the healthy siblings (Table 6). A comparison between the self-report of healthy siblings and that of the parents of the children with chronic disease showed no significant difference in physical health scores and total health scores (p>0.05), whereas the psychosocial health scores of the healthy siblings were lower than those of their parents (p<0.05).

According to the self-report of the healthy siblings, the prevalence of impaired QoL among the healthy siblings of the children with cerebral palsy, hematologic/oncologic disease, asthma, and epilepsy was 42.1%, 51.6%, 45.0%, and 30.7%, respectively, versus 14.1% among the healthy siblings of the children in the control group. The prevalence of impaired QoL was lower among the healthy siblings of children with diabetes (9.9%) and celiac disease (3.3%) than that of the healthy siblings of the children in the control group (Table 7). According to the parents’ report, the prevalence of impaired QoL among the healthy siblings of children with cerebral palsy, hematologic/oncologic disease, asthma, diabetes, celiac disease, and epilepsy was 26.9%, 10.2%, 25.8%, 14.2%, 3.0%, and 14.7%, respectively, versus 0% among the healthy siblings of the children in the control group. The parents’ report showed a lower prevalence of impaired QoL compared with that of their children’s self-report (Table 7).

An analysis of the parents’ report on children with chronic disease based on age group showed no statistically significant difference between the psychosocial health, physical health, and total health scores (p>0.05). The healthy siblings’ self-reports showed no significant differences in the psychosocial health score between age groups but a significant difference in the physical and total health scores (p<0.05), with older age groups having higher scores than the 5-7 age group.

## DISCUSSION

This study showed that the QoL is affected in varying degrees in the majority of healthy siblings of children with chronic disease. The QoL assessment of healthy siblings of 191 children with chronic disease at a median duration of 36 months following the diagnosis showed an impact on the psychosocial health, physical health, and total health scores of these siblings; these scores were significantly lower than those of the healthy siblings of healthy children. The impact was seen in both parents’ reports and healthy siblings’ self-reports. In several studies, psychosocial impact on the health sibling was observed in both the self-reports and parent reports (16,17); in contrast, there are other studies that reported no psychosocial outcomes in healthy siblings (18). Healthy siblings experience emotions such as withdrawal, aggression, depression, anxiety, guilt, and isolation, along with poor school performance and low self-esteem. As a result, they may feel lonely, ignored, excluded, neglected, and rejected (19,20,21,22,23,24). Although the majority of siblings of children with chronic disease have been negatively affected, most of these impacts are not considered psychopathological (10,25). However, whether the symptoms psychopathological or a way of coping with the situation, needs further investigation.

The factors associated with the psychosocial impact on the healthy siblings of children with chronic disease include the type and severity of the chronic disease, the time since diagnosis, and the healthy sibling’s age, gender, and coping skills (26). A severe and life-threatening disease has a larger impact on the psychological functioning of the healthy siblings, and their risk of having emotional and behavioral problems is 1.6-2 times greater than healthy children (27). The type and severity of chronic disease or disability of children may cause some effects that could be reflected in their respective siblings during adulthood (28,29). Based on the self-report of the healthy siblings, we found that the psychosocial health scores of the healthy siblings of the children with cerebral palsy, hematologic/oncologic disease, and asthma were significantly lower than that of the healthy siblings of healthy; the impact being most significant on the healthy siblings of children with cerebral palsy.

Healthy siblings of children with chronic disease may experience a loss of appetite, eating disorders, weight loss or overeating, and sleep disorders (21,22). However, the healthy children reported only a few physical symptoms when they became sick because of the severity of their sibling’s disease (21).

Combined chronic diseases are evaluated based on standard recommendations and by determining the impact of the diseases on family life rather than the specific effects and consequences of these diseases. In the present study, a comparison between the healthy siblings of children with chronic disease and those of healthy children showed that QoL scores were low; the impact was more obvious in some diseases, whereas in others, the scores were similar to those of the control group. This indicates that the diseases should be evaluated individually during QoL measurement. Studies have shown that in the presence of a sibling requiring a wheelchair, intensive care, or treatment at home/hospital (e.g., for cancer and cystic fibrosis) have a more significant negative impact on the QoL of the healthy sibling than healthy children (3,30). This may explain the negative impact on QoL of children with cancer and cerebral palsy groups. Since celiac disease only requires a diet change and no hospitalization or interventional therapies, its impact on the QoL of the healthy sibling is likely to be much lower than the healthy children. Although a previous study (3) on the healthy siblings of children with asthma have shown less impact on quality of life than the healthy children, our study showed that QoL was significantly affected in these children (3). This suggests that geographic differences may be important in QoL measurements; it also supports the hypothesis that the perception of disease may vary between different populations. Sharpe and Rossiter (25) showed that the psychosocial impact on healthy siblings of children with chronic disease with a high mortality risk (e.g., cancer) was similar to the impact on healthy siblings of children with chronic disease with a low mortality risk (e.g., diabetes, gastrointestinal diseases, and asthma). Thus, there is no clear consensus on the impact of the severity of the disease or poor prognosis on the QoL of the healthy siblings.

The time since diagnosis is likely to have an impact on the QoL of the healthy siblings as well as on the severity and prognosis of the chronic disease (19). We found that the psychosocial health, physical health, and total health scores of the healthy siblings of children with chronic disease were not associated with the duration of the chronic disease. Studies have shown that healthy siblings of children with cancer were most affected within the first month after the diagnosis, and the impact was reduced six months after the diagnosis (31). The increase in time since diagnosis led to a positive impact on the cancer patients’ siblings, but had no impact on the QoL of the healthy siblings of children with diseases such as epilepsy or diabetes (32). Some studies have shown that the impact on the healthy siblings of children with epilepsy and diabetes was reduced with an increase in age at diagnosis (33,34). Since it varies based on the type of disease, new studies are required to evaluate the impact of the duration after diagnosis on the QoL of the healthy siblings of children with chronic disease. Our evaluation based on the age groups of healthy siblings of children with chronic disease showed no difference in the parents’ report, whereas in self-report of the healthy siblings, the psychosocial health score was higher in the 13-17 and 7-12 age groups than in the 5-7 age group. The marked psychosocial impact observed in younger children indicates the importance of studies for support programs for children in this age group.

The QoL scores of healthy siblings of children with chronic disease were higher in the parents’ report than that in the healthy siblings’ self-report. This suggests that parents of children with chronic disease may overlook the impact of the chronic disease on the QoL of their healthy children. In contrast, in the parents’ report, the psychosocial health, physical health, and total health scores of the healthy siblings of children with chronic disease were significantly lower than those of the healthy siblings of healthy children. In the parents’ report, the psychosocial health scores of the healthy siblings of children with cerebral palsy, hematologic/oncologic diseases, asthma, diabetes, celiac disease, and epilepsy was significantly lower than that of the healthy siblings of healthy children; however, there was no difference in the psychosocial health scores between these conditions. In the presence of children with chronic disease, the psychosocial impact on the healthy children was perceived in a similar manner by the parents independent of the type and severity of the disease. The status of children or adolescents and their siblings has often been described through parents’ reports, which may overlook the changes in QoL of the healthy siblings, as shown in our study. Healthy siblings have usually been described by adults; however, there are substantial discrepancies between the perspectives of parents and their children. In a meta-analysis by Sharpe and Rossiter (25), the siblings’ self-reports were more positive than the parents reponse’s. Therefore, an evaluation of QoL of the healthy siblings would provide accurate information about their experiences and perceptions.

In most studies, the psychosocial assessment of healthy siblings is based on parents’ reports (18). Varni et al. (14,15) stated that a total health score <71.44 in child self-report scales and <67.44 in parent proxy-report scales indicated an “impaired QoL.” When assessing the impact on healthy children who grow up with a sibling affected by chronic disease, it is important to measure the relationship between the siblings from the healthy siblings’ perspective in addition to the QoL evaluations based on the adults’ perspective (35). In the self-report of the healthy siblings of children with chronic disease, 30.4% had an impaired QoL, which was higher than that of the healthy siblings of healthy children. In the parent reports, no QoL impairment was observed in the healthy siblings of children in the control group, but in 15.1% of the healthy siblings of children with chronic disease. The level of impaired QoL was lower in the parents’ report than that in the self-report of the healthy siblings, indicating that QoL impairment was noticed less by the parents.

Children with severe disease or disability had a more remarkable impact on the QoL of the healthy siblings than healthy siblings of healthy children. In the children’s self-reports, the prevalence of impaired QoL was 42.1%, 51.6%, 45.0%, and 30.7% in the cerebral palsy, hematologic/oncologic disease, asthma, and epilepsy groups, respectively—all higher than in the control group (14.1%). The prevalence of impaired QoL in healthy siblings of children with diabetes (9.9%) and celiac disease (3.3%) was lower than that of the healthy siblings of the children in the control group. Data on the healthy siblings of children with cancer are available; however, data on the healthy siblings of children with other chronic diseases are limited. Although there is no difference in bodily functions between the healthy siblings of cancer patients and their peers, they feel anxious about their health status (19). Murray et al. (23) reported that healthy children had a fear of developing the same disease as their siblings.

Our study has some limitations. This study aimed to evaluate the QoL of healthy siblings of children with chronic disease. However, we did not assess the disease severity for each type of disease, especially for children with cerebral palsy; this was due to the low number of children in each disease subgroup. While we enrolled children with epilepsy without neuromotor retardation, seizure frequencies might also affect the QoL of the parents and healthy siblings. Additionally, disease-related factors (especially celiac disease and diabetes) might affect the QoL; further studies are required to evaluate this.

Studies on the evaluation of QoL of the healthy siblings of children with chronic disease are limited. This is the first to include more than one type of disease and to assess the QoL from the perspective of both children and parents. Studies on healthy siblings of children with chronic disease are usually based on an evaluation of the QoL indexes of both the ill children and the healthy sibling; few studies have compared the healthy siblings of children with chronic disease and the healthy siblings of healthy children. Since different therapeutic approaches, the time since diagnosis, and the severity of disease/mortality may have a different impact on the QoL of the healthy siblings of children with chronic disease (26). Studies have shown that poor psychosocial functions observed in healthy siblings due to chronic disease or disability is also reflected during their adulthood (3,25,28,36,37). The relationship between the siblings is influenced by their cognitive, social, and emotional development, which are the determinants of QoL (38). Considering the frequency of chronic diseases and increased life expectancy, it is necessary to evaluate the impact of a child’s chronic disease on the child’s healthy siblings at home, mainly the psychosocial impact on their global QoL. The inclusion of healthy siblings in the support programs for parents of children with chronic disease may be beneficial (39,40), since brotherhood/sisterhood is a lifelong relationship that determines one’s identity and personality during adulthood.

The diagnosis of a physical chronic condition during childhood is a source of serious stress in the family and also has psychosocial impacts on the siblings of the children with chronic disease. The physical health, psychosocial health, and total health scores of healthy siblings of children with chronic disease were significantly lower than that of healthy siblings of healthy children. In healthy siblings, we observed a global impact on the HrQoL, including psychosocial scores, and a low level of parental awareness about this situation. This might increase the risk of emotional neglect and abuse of these children.

## Figures and Tables

**Table 1:**
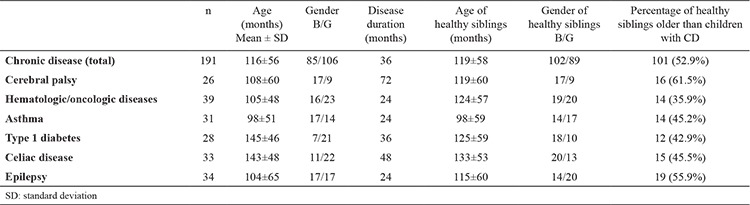
Demographic findings of children with chronic disease and healthy siblings of children with chronic disease

**Table 2:**
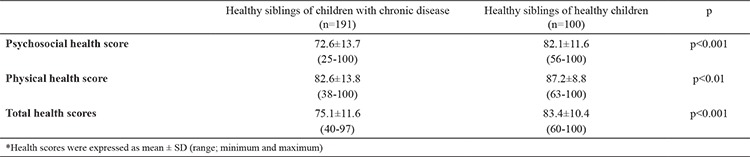
The psychosocial health, physical health, and total health scores of the healthy siblings of children with chronic disease and the healthy siblings of healthy children (self-reports of healthy siblings)

**Table 3:**
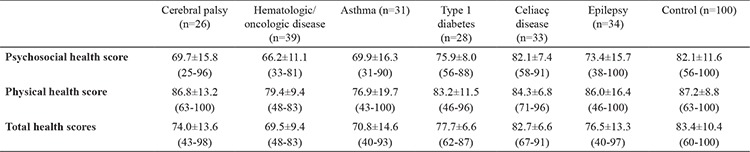
The psychosocial health, physical health, and total health scores of the healthy siblings of children in the chronic disease subgroups and the healthy siblings of healthy children (self-reports of healthy siblings)

**Table 4:**
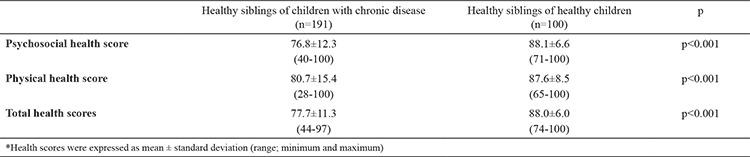
The psychosocial health, physical health, and total health scores of the healthy siblings of children with chronic disease and the healthy siblings of healthy children (parents’ report on healthy siblings)

**Table 5:**
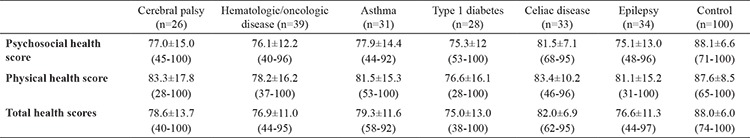
The psychosocial health, physical health, and total health scores of the healthy siblings of children in the chronic disease subgroups and the healthy siblings of healthy children (parents’ report on healthy siblings)

**Table 6:**
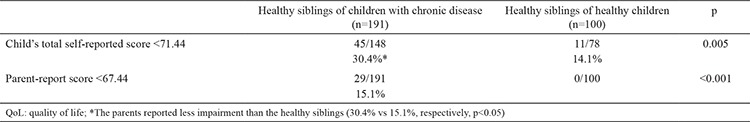
Prevalence of an impaired QoL in healthy siblings of children with chronic disease and healthy siblings of healthy children

**Table 7:**
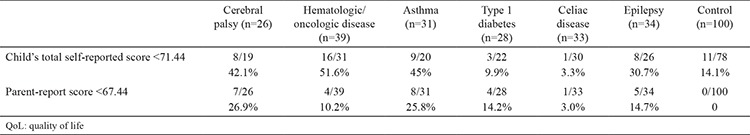
Prevalence of an impaired QoL in the healthy siblings of children with chronic disease and healthy siblings of healthy children (subgroup analysis)
